# Supplementary material for: A Decade of Lymphoma-Associated Hemophagocytic Lymphohistiocytosis: Does the Outcome Improve?
Source: J Clin Med. 2021 Oct 30;10(21):5114. doi: 10.3390/jcm10215114 (PMC8584765; doi:10.3390/jcm10215114)
Supplement: Supplementary file 1 [file jcm-10-05114-s001.zip › jcm-1442226-supplementary.pdf]

Supplemental Table S1. Clinical information in patients undergoing allo-HSCT.

	Total	B-cell lymphoma (n = 3)	T-cell lymphoma (n = 3)
**Status before allo-HSCT, *n***			
CR	1	1	0
PR	4	1	3
PD	1	1	0
**Response post allo-HSCT, *n***			
CR	3	1	2
PR	2	1	1
Not evaluable	1	1	0
**Conditioning regimen, *n***			
MAC	3	1	2
RIC	3	2	1
**Transplant-related mortality, *n***	1	1	0
**Donor type, *n***			
MSD	4	2	2
MUD	1	0	1
MMUD	1	1	0
**Current status, *n***			
Survival	3	1	2
death	3	2	1

allo-HSCT: allogeneic hematopoietic stem cell transplantation. CR: complete remission; PR: partial response; PD: progressive disease. RIC: reduced intensity conditioning; MAC myeloablative conditioning. MSD: matched sibling donor; MUD: matched unrelated donor; MMUD: mismatched unrelated donor

Supplemental Table S2. Chemotherapy regimen comparisons among HLH patients diagnosed between 2004–2012 and 2013–2021.

	Total		Year of diagnosis 2004–2012		Year of diagnosis 2013–2021		p value
**CHOP-like**	22	(48.9%)	9	(34.6%)	13	(68.4%)	0.150
**CHOEP**	10	(22.2%)	7	(26.9%)	3	(15.8%)	
**ESHAP**	6	(13.3%)	5	(19.2%)	1	(5.3%)	
**Others**	7	(15.6%)	5	(19.2%)	2	(10.5%)	

HLH: hemophagocytic lymphohistiocytosis

This result is analyzed by the Chi-square test. All data are shown as number and percentage

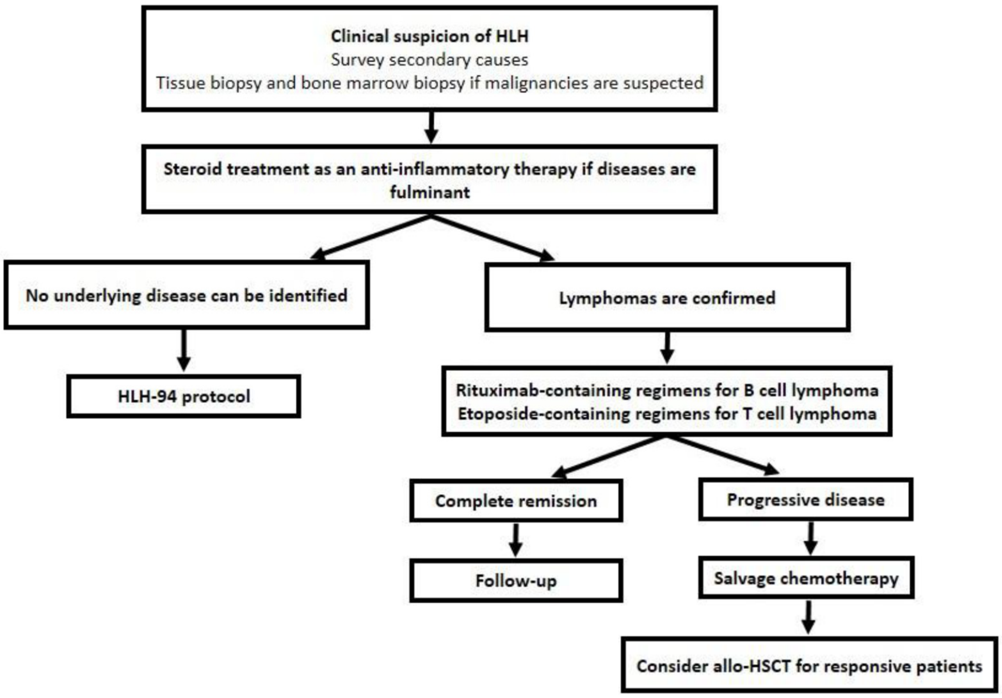

HLH: hemophagocytic lymphohistiocytosis
Allo-HSCT: allogeneic hematopoietic stem cell transplantation

Supplemental Figure S1. Therapeutic algorithm of patients with HLH.
